# Case Report: TAVI in a Patient with Single Coronary Artery and Bicuspid Valve

**DOI:** 10.1155/2020/2569506

**Published:** 2020-01-24

**Authors:** Murielle Bertin, Mohamed Aboukofa, Pierre Francois Laterre, Zuhair Yousif

**Affiliations:** ^1^Department of Anaesthesia, Mediclinic City Hospital, Dubai Health Care City, Building 37, Dubai, UAE; ^2^Intensive Care Department, Saint Luc University Hospital, UCL, Avenue Hippocrate 10, 1200 Brussels, Belgium; ^3^Department of Cardiology, Mediclinic City Hospital, Dubai Health Care City, Building 37, Dubai, UAE

## Abstract

A single coronary artery is a rare congenital abnormality which consists of one coronary artery arising from the aortic trunk by a single coronary ostium and providing the perfusion of the entire myocardium. Its prevalence is approximately 0.024-0.066% of the population undergoing coronary angiography. A bicuspid aortic valve is the most common congenital cardiac abnormality and is found in 0.4-2.25% of the population. The coexistence of these two abnormalities together with severe aortic stenosis is extremely rarer. We report a patient who underwent transcatheter aortic valve implantation (TAVI) with a single coronary artery and a bicuspid valve. The procedure was successful, and the patient was discharged home without complication. To the best of our knowledge, this is the first report of these combined abnormalities and TAVI.

## 1. Introduction

A single coronary artery is at a high risk of coronary obstruction during a TAVI procedure. The bicuspid aortic valve is also at a high risk of coronary obstruction. In addition, there is an increased risk of paravalvular leakage and the need for operative pacemaker device placement.

Patients with bicuspid aortic valves have previously been excluded from clinical trials involving TAVI, and this abnormality continues to be a relative contraindication for the use of TAVI.

However, a recent success in the placement of new-generation valves means that a bicuspid aortic valve is no longer a contraindication to TAVI and this technique may offer a viable alternative to open-heart surgery in the elderly population.

## 2. Case Presentation

A 65-year-old woman with severe, symptomatic aortic stenosis was admitted to a hospital for a TAVI. Her past medical history included type 2 diabetes mellitus, hypertension, diabetic chronic kidney disease, and dyslipidemia. The patient was well known to have a bicuspid aortic valve and had been followed up by echocardiography for 20 years. She complained of exertional dyspnea for the last three months and was admitted with severe pulmonary edema. Following careful assessment, it did appear that the primary precipitant for refractory pulmonary edema is severe aortic stenosis. The aortic valve area of a heavily calcified valve was only 0.3 cm^2^. Aortic valve replacement was a necessary therapeutic strategy to achieve hemodynamic stability and reduce in-hospital mortality.

Contrast-enhanced computed tomography (CT) scan was contraindicated because of her renal impairment. Noncontrast computed tomography would not have added significant diagnostic information to influence the treatment plan. Catheterization with limited contrast injections for coronaries, aortic root, and peripheral bed had provided the required details to plan the procedure with less contrast than computed tomography with contrast. In addition, noninvasive imaging with echocardiography has been used pre and postprocedure.

**Figure 1:**
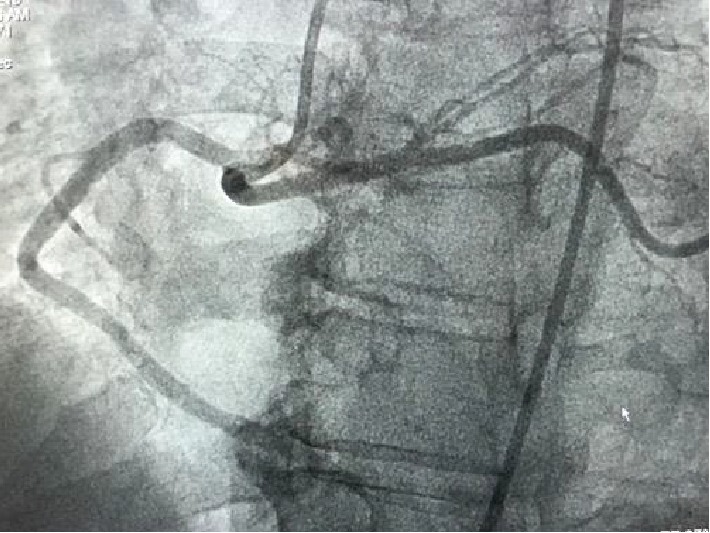
Single coronary artery with the left main coronary artery coming from the right coronary artery.

Coronary angiography was normal but revealed a single right coronary artery, with the left main coronary artery arising from the proximal right coronary artery (Figure 1). This is a very rare congenital malformation which can be a lethal obstruction of the orifice of the right coronary artery occurring secondary to stenosis or because of coronary emboli during the deployment of a new aortic valve.

**Figure 2:**
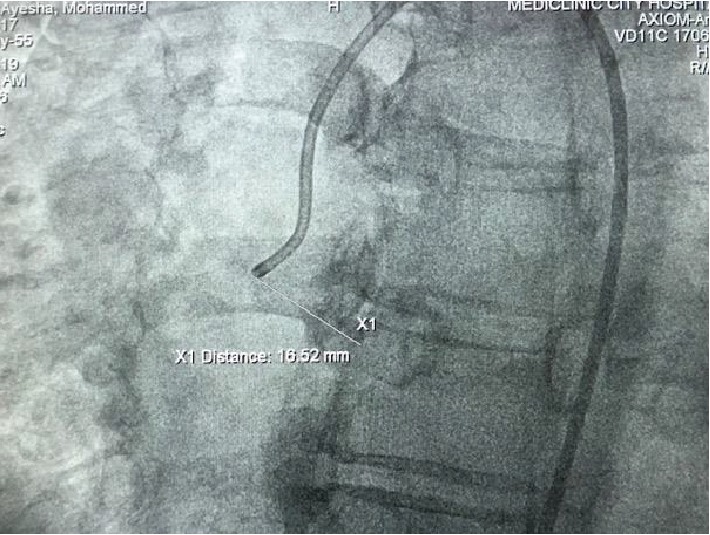
Calculation of the distance between the single coronary artery and the aortic valve.

The distance of the single coronary artery to the bicuspid aortic valve was >12 mm and to the annulus of the valve was >20 mm, and to S5 was 25 mm (Figure 2).

**Figure 3:**
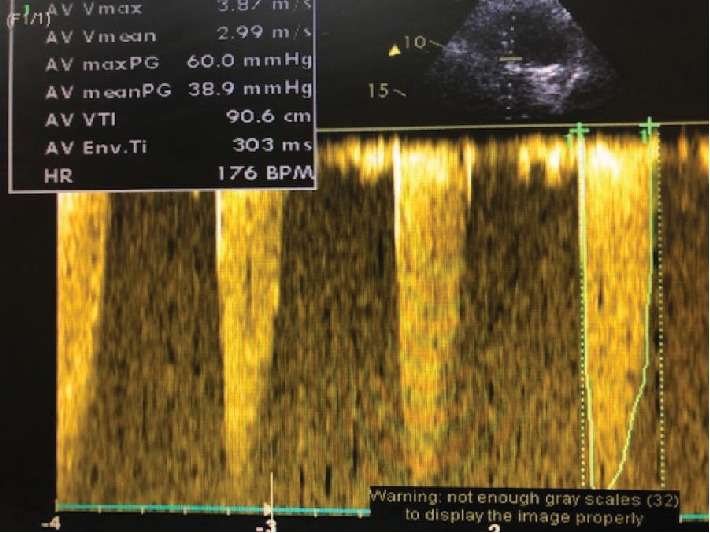
Echography done just before TAVI.

Echocardiography showed a severe bicuspid aortic valve stenosis with a gradient of 80/50 and a valve area of 0.3 cm (Figure 3). Mild mitral regurgitation was observed, but there is no aortic regurgitation.

An electrocardiogram showed a normal sinus rhythm.

The patient was informed by the cardiac team, and it was agreed to proceed with an urgent TAVI procedure.

**Figure 4:**
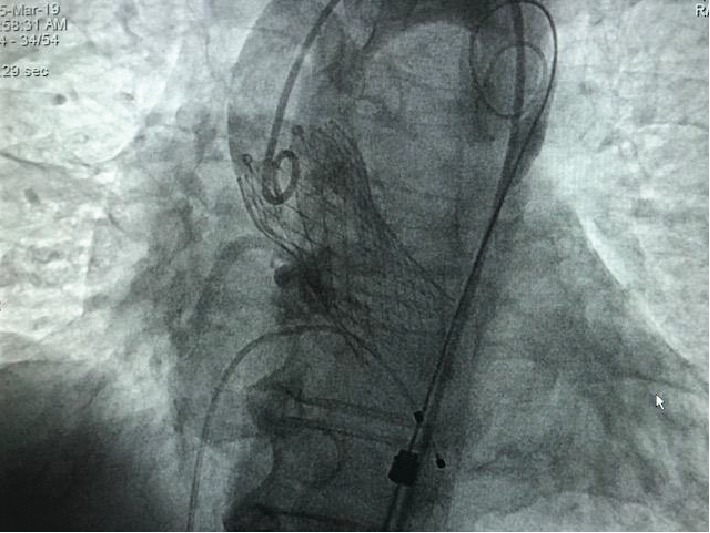
26 mm Evolut Pro valve in place.

A direct implantation of Evolut Pro, 26 mm valve from Medtronic, was performed using a right femoral artery approach. Close monitoring of the single coronary artery with coronary angiography revealed no complications (Figure 4). The left main coronary artery took its origin from the proximal right coronary artery. 2Proglide was used on the right-hand side, with 6F Angioseal on the left side; a hundred mL contrast medium was used.

The procedure was performed in the cardiac catheter laboratory under local anaesthesia plus sedation, and the patient remained hemodynamically stable throughout.

The patient was then transferred to the intensive care unit for observation and was discharged to the ward the following day. The patient remained in sinus rhythm and did not require a pacemaker postoperatively.

The post TAVI echocardiography showed a well-functioning aortic valve with trivial paravalvular leakage and no pericardial effusion. Ejection fraction was 70% with a peak and mean gradient after TAVI of 10/4. The mild mitral valve regurgitation apparent before the procedure remained unchanged.

The patient was asymptomatic and consequently discharged home after 3 days. The two-week follow-up showed a well-functioning valve with similar echocardiography. The electrocardiogram confirmed normal sinus rhythm, and the patient remained asymptomatic.

## 3. Case Discussion

Single coronary artery is a very rare congenital artery anomaly. It is found in approximately 0.03 to 0.05% of the population undergoing coronary angiography [1].

Lipton and coworkers propose a very useful angiographic classification and describe “R” as the right type and “L” as the left type according to the site of origin of the single coronary artery in the right or left sinus of Valsalva [2].

The etiology of a single coronary artery is unclear. This anomaly can be asymptomatic or may lead to angina, syncope, myocardial infarction, cardiac arrhythmia, congestive heart failure, or sudden death in case of stenosis or blockage of the only coronary artery ostium.

In the case of TAVI, valve deployment should be extremely cautious because any obstruction of this only coronary artery could be proven fatal.

Another challenge for the operator in this case was the bicuspid aortic valve as it resulted in a smaller opening with heavy calcification which bulged down close to the interventricular node. Hence, passing the wire from the femoral artery through the valve took longer time and was more technically challenging and the procedure could be expected to be more proarrhythmogenic than usual. In cases of the bicuspid aortic valve, the literature commonly reports the need for insertion of a permanent pacemaker post-TAVI [3].

The asymmetric nature of the bicuspid valve orifice and heavy regional calcification has been a challenge for adequate valve positioning and expansion for TAVI operators. There is an increased risk of coronary occlusion, aortic dissection, and annular rupture in these patients.

A careful evaluation of the aorta is compulsory as a bicuspid aortic valve is often associated with coarctation of the aorta, aortic dissection, and aortic aneurysm. These aortic pathologies may necessitate surgical intervention, rather than TAVI.

An additional challenge for the operator is correct valve selection. Nowadays, the use of a CT angiography may improve the selection of the valve size and device [4]. This minimizes the risk of complications including aortic annulus rupture, perivalvular leak, and coronary occlusion.

A large study comparing CT scans of bicuspid and tricuspid valves showed that aortic valve ellipticity indices were smaller in patients with bicuspid valves (1.24 vs 1.29) but the annular area was larger (5.21 vs. 4.63 cm^2^) than that with tricuspid valves. We know that more calcification of the annulus and eccentric leaflets in the bicuspid valve give a higher chance of paravalvular leakage and higher chance of the need for pacemaker implantation after TAVI.

Compared to the tricuspid valve, TAVI operators will pay extra attention to the angiographic visualization of the annulus level and selection of proper projection for implantation of the valve. There is a risk of elliptic distortion or noncircular expansion, and it seems that risks are less when the valve is placed deeper below the annulus level than exactly at the level of the annulus. Self-expanding valves seem safer and are associated with less paravalvular leakage when compared to balloon mounted prostheses.

There are different classifications of the bicuspid valve, and the most widely used is that of Sievers and Schmidtke [5], based on the number of cusps, presence of raphes, and the position and symmetry of the cusps.

There is a relatively higher proportion of patients requiring pacemaker implantation after TAVI if they have bicuspid aortic valves.

Risk factors for coronary obstruction are low origin of the coronary arteries, bulky calcifications, a small sinus of Valsalva and valve misplacement, or coronary emboli.

Patients so afflicted will present with severe hypotension immediately after the balloon valvuloplasty or bioprosthesis implantation.

In previous reports of single coronary artery with TAVI, operators performed angiography during balloon valvuloplasty to anticipate the risk of coronary obstruction [6]. In one case, the operator placed a coronary wire to serve as a protection of the single coronary during balloon valvuloplasty and bioprosthesis implantation [7].

Another team did not perform any protective intervention during TAVI for the single coronary but used a self-expandable valve because of the recapturable property of the valve.

A Medtronic Core Valve can be recaptured up to 2/3 of the bioprosthesis deployment. In the case of any complication during TAVI, e.g., coronary obstruction, the valve can then be recaptured by the operator [8].

In our case, the operator spent time localizing precisely the single coronary artery origin before positioning and deploying the new valve very cautiously. The position of the valve was carefully adjusted, guided by intraoperative imaging with aortic root injections. The ample cusp to coronary ostia distance did not mandate the need for dedicated coronary protection procedures. There was no need to recapture the new valve which was perfectly positioned on the first attempt. No arrhythmia occurred, and no pacemaker was required. Very good valve functioning with only a trivial leakage was demonstrated on the echocardiogram performed immediately after the placement of the Medtronic valve. The patient was stable hemodynamically and did not require any inotropic support.

All data generated or analyzed during this study are included in the case presentation of this article: Figures 1–4.

## 4. Conclusion

We describe here a successful case of TAVI in a patient presenting with a combination of two rare abnormalities: a single coronary artery and a bicuspid aortic valve. The coexistence of a single coronary artery with severe aortic stenosis is extremely rare. The most feared complication of TAVI is a lethal obstruction of the single coronary artery origin. There are limited reports in the literature of TAVI associated with single coronary artery and no case of a bicuspid valve with a single coronary artery and TAVI. To our knowledge, this is the first reported case of TAVI with a self-expandable bioprosthesis. The procedure used the new Medtronic valve which can be recaptured in case of misplacement.
